# Hearing Loss and Cognition: What We Know and Where We Need to Go

**DOI:** 10.3389/fnagi.2021.769405

**Published:** 2022-02-28

**Authors:** Danielle S. Powell, Esther S. Oh, Nicholas S. Reed, Frank R. Lin, Jennifer A. Deal

**Affiliations:** ^1^Department of Health Policy and Management, Johns Hopkins Bloomberg School of Public Health, Baltimore, MD, United States; ^2^Cochlear Center for Hearing and Public Health, Johns Hopkins Bloomberg School of Public Health, Baltimore, MD, United States; ^3^Division of Geriatric Medicine and Gerontology, Johns Hopkins University School of Medicine, Baltimore, MD, United States; ^4^Department of Epidemiology, Johns Hopkins Bloomberg School of Public Health, Baltimore, MD, United States; ^5^Department of Otolaryngology, Johns Hopkins University School of Medicine, Baltimore, MD, United States

**Keywords:** hearing loss, cognitive decline, dementia, prevention, dementia management, sensory loss, risk factor

## Abstract

Although a causal association remains to be determined, epidemiologic evidence suggests an association between hearing loss and increased risk of dementia. If we determine the association is causal, opportunity for targeted intervention for hearing loss may play a fundamental role in dementia prevention. In this discussion, we summarize current research on the association between hearing loss and dementia and review potential casual mechanisms behind the association (e.g., sensory-deprivation hypothesis, information-degradation hypothesis, common cause). We emphasize key areas of research which might best inform our investigation of this potential casual association. These selected research priorities include examination of the causal mechanism, measurement of co-existing hearing loss and cognitive impairment and determination of any bias in testing, potential for managing hearing loss for prevention of dementia and cognitive decline, or the potential to reduce dementia-related symptoms through the management of hearing loss. Addressing these research gaps and how results are then translated for clinical use may prove paramount for dementia prevention, management, and overall health of older adults.

## Introduction

Hearing loss is the leading potentially modifiable risk factor for dementia; up to 8% of global dementia cases are estimated as being attributable to hearing loss (Livingston et al., 2017, 2020). Following foundational initial research from over 5 decades ago, recent years have seen a steep rise in scientific investigation of the hearing-dementia association. As far back as 1968, participants demonstrated poorer performance on word-recall cognitive tasks in an experimental condition designed to mimic hearing loss and decreased speech intelligibility compared to normal listening conditions (Rabbitt, 1968). Two decades later, a seminal case-control study (Uhlmann et al., 1989) reported greater odds of dementia in those with hearing loss compared to normal hearing.

Even with our expanding knowledge of the association between hearing loss and dementia, significant gaps in our understanding persist. In this article, we aim to (1) provide a foundational understanding of hearing loss presentation and diagnosis; (2) review current hearing loss epidemiology (3) provide context to the existing research on the relationship between hearing loss and dementia to guide dementia prevention and intervention recommendations or research; (4) discuss the leading theories on the pathways behind the hearing-dementia association; and (5) present four crucial areas for future research. With the demographic changes in the coming decades, we conclude with calls for inter-disciplinary collaboration and posit a unique opportunity to alter the landscape of cognitive aging and dementia care. Understanding the role and presentation of hearing loss in the adult population allows for better appreciation of where the field of auditory science may fit into a broader picture of dementia prevention and interventions.

## Hearing Measurement and the Presentation of Hearing Loss

### Measuring Hearing Ability

**FIGURE 1:**
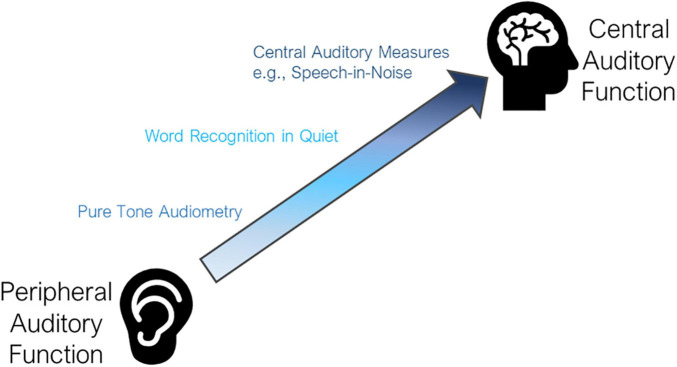
Levels of auditory function. The auditory system spans between peripheral and central auditory function, gaining complexity and higher cognitive processing as you activate more central auditory function. Pure tone audiometry is the most common metric for measurement of peripheral function. Word recognition (i.e., repeating back a single word heard without the use of visual cues) requires additional input of central auditory function. Central auditory measures such as speech-in-noise testing are the most complex and objective measures of central auditory function commonly performed.

The act of hearing involves two inter-related processes and components of the auditory system – the peripheral auditory system and the central auditory system (Musiek and Baran, 2018) and additional non-auditory influences such as cognitive processing, education, situational influences, and self-perception, among others (Figure 1). Yet, both parts of the auditory system must work in tandem for an individual to appropriately detect and understand a sound. This process is essential for auditory interaction with the environment and is instrumental for quality communication. Yet most commonly, it is the peripheral auditory system that is considered in discussion of hearing loss. The few epidemiologic studies that include a formalized measure of hearing primarily consider peripheral hearing ability alone. Moreover, many epidemiologic studies rely on the self-reported measures of hearing. While measures of self-report hearing have their utility, self-report hearing is complex and represents perceived functional ability of hearing, incorporating aspects of the peripheral and central auditory system as well as the social environment, psychosocial aspects, and listening demands.

#### Peripheral Auditory Function

The peripheral auditory system includes the outer ear (pinna), ear drum (tympanic membrane), middle ear bones (malleus, incus, and stapes), and cochlea (Musiek and Baran, 2018). These components work together to transform auditory sound waves or acoustic energy of the surrounding environment captured by the outer ear and ear drum into mechanical energy in the middle ear space, and, eventually, encoding information as an electrical signal in the cochlea to be sent along the central auditory system to the brain (Musiek and Baran, 2018). While a discussion of the complete anatomy and physiology of the peripheral auditory system is beyond the focus of this article, an individual’s ability to detect the presence of auditory stimuli initiates within and is dependent upon the peripheral system.

**FIGURE 2:**
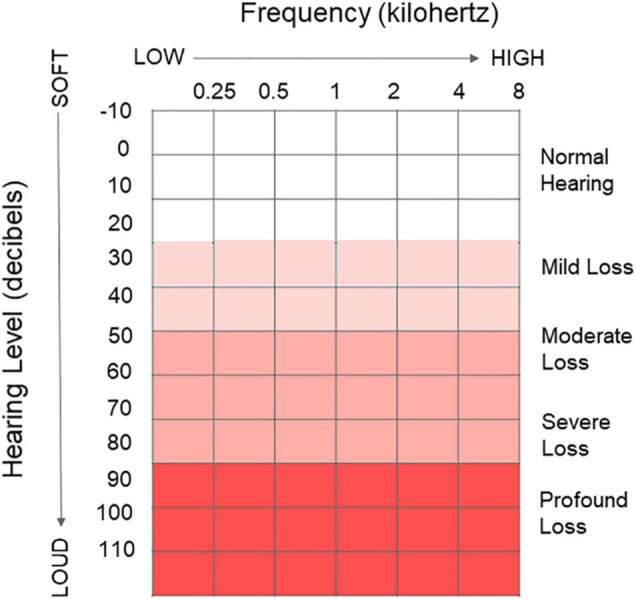
Audiogram. Graphical clinical tool used to depict peripheral hearing ability and record peripheral hearing thresholds as measured via pure tone audiometry. *X* axis represents the frequency (kHz) of sound going from low frequency to high frequency 0.25–8 kHz. *Y* axis represents the volume of the presented pure tone, going from very soft to very loud (–10 to 110 dB). Common clinical categories to describe hearing loss are indicated ranging from normal hearing to a profound hearing loss. The lowest volume at each frequency an individual indicates they can hear the tone is recorded for each ear on the graph.

The most common clinical tool for measurement of peripheral hearing acuity in adults is pure tone audiometry (Katz, 2015), with results graphically recorded on an audiogram (Figure 2). This gold standard for testing is performed in a sound-proof booth using calibrated headphones. An auditory stimulus (i.e., pure tone) is presented at particular frequencies [measured in Hertz (Hz)] commonly within the range of 250–8000 Hz. The presentation level of each pure tone begins at an audible listening level for each individual and is decreased to ascertain the lowest threshold level [i.e., the volume in decibels hearing level (dB HL)], at which the individual indicates detection of the tone. In the epidemiologic literature, results are often summarized using the World Health Organization criteria as a four-frequency pure tone average (PTA), or the average of responses at 500, 1000, 2000, and 4000 Hz (Katz, 2015). Hearing loss may also be discussed within common clinical categories (i.e., normal [PTA < 20 dB], mild [20 to <35 dB], moderate [35 to <50 dB], moderately severe [50 to <65 dB], severe [65 to < 80 dB], or profound [80 dB or greater]) (World Report on Hearing, 2021) or other categorizations (Clark, 1981) which are not currently adhered to in clinical audiology.

#### Central Auditory Function

Following passage through the peripheral auditory system, the electrical signal created by the cochlea is sent to the auditory nerve where it is then decoded by the brain (Musiek and Baran, 2018; Figure 2). Central hearing ability is therefore dependent upon the integrity of the auditory signal passed from the peripheral auditory system as well as additional cognitive processing (Gates, 2012; Humes et al., 2012). While peripheral hearing ability may be simplified as the ability to detect sound, central hearing ability then is the ability for the brain to understand and make sense of environmental sounds, requiring significant higher-level processing (i.e., identifying signals of interest from ambient noise, giving spatial awareness, adding together several pure tone signals to create complex signals such as speech, etc.).

Measurement of central auditory function is often completed through the presentation of speech within the presence of increasing volume of selected types of background noise (i.e., speech-in-noise testing) or specialized tests of central auditory processing ability that involve specific tasks like listening to degraded or temporally modified speech (Gates, 2012). However, this testing is far less frequently completed in either adult clinical visits or large-scale epidemiologic studies as compared to smaller research studies. Measurement tools for central auditory function transcends the auditory pathway with varied emphasis on how much peripheral auditory function has on performance. Measures might include the presentation of carefully selected single words via tests of word recognition, or presentation of sentences which additionally might provide context clues for word identification either in quiet or increasing levels of background noise. While central hearing ability encompasses more than speech-in-noise ability as noted, current clinical audiologic testing primarily considers basic speech-in-noise ability and not necessarily higher-level auditory cognitive processing tasks [i.e., the differentiation of sound sources within a complex environment (Bregman, 1993)]. However, tests of auditory cognitive ability designed to account for general cognitive ability or specific cognitive domains, in addition to peripheral hearing ability, have been developed (Goll et al., 2010, 2011, 2012; Grube et al., 2016) yet are in limited clinical use. It is conceivable these targeted auditory cognitive tests, with further study and if adopted more widely, may aid in understanding of the connection between neurobiological and specific central auditory ability and dementia subtypes and 1 day be included within clinical neurological work-ups as an additional indicator or early-stage dementia. However, until differentiation is feasible, the interdependence between central auditory function and cognitive processing blurs the distinction between the two processing abilities. In addition, heterogeneity in prior study has created significant barriers to pooled evidence and causal inference regarding the role of central hearing ability within cognitive impairment and testing. While more detailed description is beyond the scope of this article, a functional understanding of both aspects of the hearing system is important when considering dementia prevention strategies.

### The Presentation of Age-Related Hearing Loss

Age-related hearing loss (ARHL), also known as presbycusis, accounts for the largest percentage of hearing loss cases around the world and presents as a gradual decrease in hearing ability. The outer hair cells and other sensory cells in the cochlea are progressively damaged and unable to regenerate (Pickles, 2013). The result is the impaired encoding of sound, decreased precision, and a distorted auditory signal sent to the brain. This form of hearing loss commonly leads to decreased detection of the higher frequency sounds of speech before lower frequency sounds. The result is reduced ability to hear high-frequency consonants (i.e., /s/, /f/, th/), the parts of speech that provide crispness and clarity. In turn, many with ARHL do not necessarily report difficulty with volume of speech but instead indicate speech sounds muddled or garbled, especially when in the presence of background noise. For everyday function, this may lead to difficulty communicating or interacting with others depending on the listening environment. Those with ARHL may have difficulty hearing or understanding what others are saying in a crowded or noisy setting, such as a restaurant. They may also have difficulty hearing and understanding the television, on the phone, when someone speaks from another room or with their back turned.

The impact of a given pattern of hearing function as represented on the audiogram on communicative performance may vary widely from individual to individual (George et al., 2007; Chang et al., 2009). An individual’s functional performance with their hearing depends on not only their peripheral hearing (audiogram) but also their central hearing ability (i.e., central auditory processing, speech-in-noise performance), personal motivation, self-efficacy, listening environments and demands. The availability of cognitive resources to help support interpretation of a degraded auditory signal may also have a significant role in individual experience.

## Epidemiology of Hearing Loss

### The Prevalence and Cost of Hearing Loss

**FIGURE 3:**
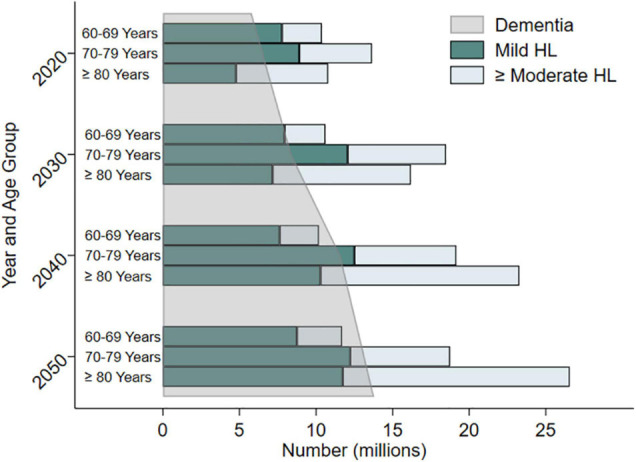
Estimated and projected trends in prevalence of hearing loss (mild hearing loss and moderate or greater hearing loss) and dementia in older adults by age categories in the United States from 2020 to 2050. Data compiled from Goman et al. (2017) and the Alzheimer’s Disease Facts and Figures 2020 Report. Adapted from Powell et al. (2021).

Hearing loss currently impacts an estimated 20% of the global population, or more than 1.5 billion people (World Report on Hearing, 2021). By 2050, estimates now project nearly 2.5 billion people with hearing loss, such that nearly 1 in 4 individuals will have some degree of loss (World Report on Hearing, 2021). The breakdown in hearing loss prevalence by age category varies by country, with Western countries demonstrating greater prevalence at older ages. In the United States, over two-thirds of adults age 70 years and older have a bilateral hearing loss (i.e., hearing loss in both ears) (Lin et al., 2011; Goman and Lin, 2016; Figure 3). It was estimated over 44 million older adults would have hearing loss in 2020 with expected increase to over 73 million by 2060 (Goman et al., 2017). However, analysis in a nationally representative sample of older adults suggests that while the overall prevalence of hearing loss is increasing due to greater numbers of older adults, the age-specific prevalence of hearing loss in the United States appears to be decreasing over the last decade, by about 2% in adults compared to the prior decade (Hoffman et al., 2017). This age-specific decrease may be related to improvements in health care or access to care and prevention of known risk factors for hearing loss, improved education, environmental noise protections [e.g., workplace hearing protection, time limits for noise exposure, community noise level limits], and management of vascular or disease risk factors.

Globally, the cost of unaddressed hearing loss is estimated at over $980 billion annually (World Report on Hearing, 2021). Economic costs of lost productivity due to hearing loss is estimated widely at $1.8–$194 billion in the United States alone. Excess medical costs due to hearing loss was estimated at $3.3–$12.8 billion (Huddle et al., 2017). Individually, over a span of 10 years, the difference in mean total healthcare costs for someone with hearing loss compared to someone without hearing loss is over $22,000 in the United States (Reed et al., 2019), due in part to increased rate of hospitalizations, higher risk of hospital readmission, and longer hospital stays. This economic cost may take a toll on societies and families managing the social, economic, and healthcare needs of older adults. While hearing loss has its greatest prevalence among older adults, significant hearing loss is not necessarily an inevitable consequence of aging, as everyone has a different hearing trajectory shaped by genetic, environmental, and health related factors from across the life course.

### Risk Factors for Hearing Loss

The prevalence of hearing loss varies by non-modifiable factors such as certain identified syndromes and genetic factors (World Report on Hearing, 2021). Additional non-modifiable factors include race/ethnicity and possibly biological sex (Curhan et al., 2017). While a lower prevalence of hearing loss is noted among women compared to men, thought primarily related to behavioral and lifestyle choices (i.e., increased noise exposure), some evidence suggests a biological protective effect of estrogen among women (Hultcrantz et al., 2006; Shuster et al., 2019). Additionally, hearing loss has a lower prevalence in Black adults compared to Hispanic or non-Hispanic White adults (Bainbridge and Wallhagen, 2014; Goman and Lin, 2016), suspected in part due to a protective effect of melanin in the inner ear (Lin et al., 2017).

Additional potential causative risk factors for hearing loss across the life course have also been identified (World Report on Hearing, 2021). While many risk factors for hearing loss stem from prenatal or perinatal causes/determinants (i.e., intrauterine infections, delivery complications), other risk factors for hearing loss in adolescence and adulthood have also been identified (World Report on Hearing, 2021). These include: otitis media (i.e., ear infections), viral infections or pathogens (e.g., measles, mumps, meningitis, HIV, Lassa virus, Ebola), chronic disease (e.g., hypertension, cardiovascular disease, diabetes), smoking, use of ototoxic medications (i.e., cisplatin, carboplatin, gentamicin) head trauma, excessive noise exposure from occupational, recreational, or environmental noise, ear-related conditions such as Meniere’s disease, autoimmune disease, or vestibular schwannomas, and nutritional deficiencies from nutrients like Vitamin A, zinc, and iron. As stated, one of the greatest risk factors for hearing loss is increasing age due to age-related sensorineural hearing loss. This is a multifactorial condition thought influenced by genetics, environmental factors, lifestyle, and illnesses. While it is not yet determined if all of these factors are causal, each is correlated with increased risk of hearing loss.

### Modifiability of Hearing Loss

Historically, hearing loss has been considered a benign component of the aging process resulting primarily in impaired communication. However, research over the last few decades has highlighted hearing loss as a precipitating factor for additional functional and neuropsychiatric disorders in older adults and has spurred changes in thought within the medical and public health community (Lopez et al., 2011; Lin et al., 2013; Kamil et al., 2014). Importantly, hearing loss is generally considered modifiable through amplification (i.e., hearing aids or amplification devices). The overall goal of amplification is to aid in the restoration of auditory input from impaired peripheral hearing and serve as a tool to help manage the presence of background or competing noise for communication and comfort. Therefore, the scientific and clinical community has begun to recognize the potential protective benefit of hearing loss management, particularly for neuropsychiatric conditions like depression and dementia (Rutherford et al., 2018). Therapeutic opportunity exists, as hearing aids are vastly underutilized: on average only ∼30% of eligible adults obtain hearing aids in the United States (Bainbridge and Wallhagen, 2014), with similar uptake reflected in peer countries (World Report on Hearing, 2021). Recent investigation suggests trends in improved uptake of amplification devices in the United States, however disparities and divergences are noted for lower income older adults (Reed et al., 2021). While significant barriers and disparities in hearing health care and accessibility of care and services remain, as will be discussed later, the potential room for intervention growth and public health benefit is substantial. Management of hearing loss through amplification options may not only decrease risk for neuropsychiatric conditions (Rutherford et al., 2018), but may also improve treatment adherence, patient satisfaction, and improve overall health-related quality of life for older adults (Bigelow et al., 2020). Understanding subgroups of older adults who may receive particular benefit from increased amplification use may therefore save health care costs, reduce burden, and improve outcomes – including dementia.

### Dementia Prevention Through Hearing Loss

The expected rise in dementia cases around the world requires action on dementia prevention and intervention across innovative and multi-disciplinary channels to meet the pressing societal and health needs. With no known dementia cure, epidemiologic research then seeks to discern risk factors associated with the progression of dementia pathology, progression of dementia symptoms, or with dementia diagnosis to delay or minimize the impact of clinical symptoms (Livingston et al., 2020). If we can identify such risk factors, we may cultivate increased focus and direction for research to possibly delay dementia onset. Delaying the onset of dementia by just 5 years through intervention could lead to a 57% reduction in the number of dementia cases (Sperling et al., 2011) and 40% lower cost for care and services in 30 years (Zissimopoulos, 2014). Epidemiologic research has provided much of the foundational evidence for the association between hearing loss and dementia. This research has allowed for recognition of how the under-utilization of hearing loss treatment strategies presents an opportunity for potential advancement. With potential collaboration across the unique disciplines associated with cognitive aging and auditory science, opportunity for novel intervention and prevention strategies to enrich our evidence base for dementia prevention exists.

## Linking Hearing Loss and Dementia– Current Knowledge

In this review, we highlight evidence of the association and potential causal effect primarily within studies utilizing completed objective measures of auditory function (i.e., audiogram, validated speech-in-noise testing). While consideration of self-reported hearing is important, it integrates aspects of not only peripheral auditory function but also of an individual’s perceived hearing and communication ability, incorporating environmental and communication demands, mental health, listening motivation and expectations, and central auditory function.

Heterogeneity in study methodology and design creates challenges to synthesize data from hearing and cognition research. Heterogeneity exists across audiometric and cognitive parameters as well as sample sizes, populations studied, and consideration of confounders, to name a few. Within auditory science, heterogeneity includes aspects such as differing means to define hearing loss, pure tone frequencies used, or how hearing ability is categorized. For cognitive research, variability in the neurocognitive tests used, domains investigated, utilization of cognitive screeners, patient or physician report, and ways of defining cognitive change (i.e., cognitive decline, subjective cognitive impairment, mild cognitive impairment, or diagnosed dementia) all create obstacles for evidence synthesis.

### Peripheral Auditory Function

The *Lancet* Commission (Livingston et al., 2017, 2020) reported a pooled relative risk of 1.9 times greater risk of incident dementia among hearing impaired individuals 55 years of age and older compared to those with normal hearing. However, only three longitudinal studies (Lin et al., 2011; Gallacher et al., 2012; Deal et al., 2016) on the association between peripheral hearing impairment and incident dementia met the Commissions specified inclusion criteria of audiometrically (objective) measured hearing, longitudinal evaluation (at least 5 years), and covariate adjustment. While requirement of longitudinal evaluation and covariate adjustment require little justification, audiometrically measured hearing allows for measurement of a more isolated effect of peripheral hearing loss due to cochlear damage rather than the more convoluted measurement of central auditory function or self-report hearing. As audiometry serves as the gold-standard for objective measures of hearing, its validity has been well investigated in a variety of populations. As audiometry is a measure of peripheral function, it is likely not strongly influenced by cortical changes from dementia and can be appropriately completed within populations even with early dementia (Uhlmann et al., 1989; McClannahan et al., 2021). These aspects support minimal concern of the association resulting from reverse causation with dementia leading to impaired audiometry and PTA, although other studies suggest differing conclusions (Brenowitz et al., 2020). Detailed summaries of current evidence have been provided in a handful of other publications (Albers et al., 2015; Fortunato et al., 2016; Thomson et al., 2017; Loughrey et al., 2018; Yuan et al., 2018; Sharma et al., 2021).

A previous meta-analysis (Loughrey et al., 2018) found the odds of cognitive impairment for those with age related hearing loss was 1.22 (95% CI: 1.09, 1.36) and odds of dementia was 1.28 (95% CI: 1.02, 1.59) times higher than those with normal hearing. Prior work has noted an association between hearing loss and declines in global cognitive function, executive function, processing speed, and memory (Lin et al., 2011; Loughrey et al., 2018; Alattar et al., 2020; Gao et al., 2020; Brewster et al., 2021). However, what constitutes hearing ability or cognitive impairment often differs across studies. Some studies have included continuous measures of hearing as decibels hearing level (dB HL) (Armstrong et al., 2019), while others have categorized hearing ability by clinically recognized categories [i.e., mild or greater loss (Lin et al., 2011, 2013; Deal et al., 2016; World Report on Hearing, 2021)] or have only considered specific frequency ranges (Uhlmann et al., 1989; Gates et al., 1996) as compared to the common clinically utilized range of 500–4000 Hz or 500–8000 Hz. The diverse definitions of hearing loss make it challenging to synthesize the current evidence base as the degree of hearing difficulty varies by categorization used and represents different levels of resultant impairment and functional ability. For example, categorization of those with *any* measured hearing loss (i.e., Pure Tone Average [PTA] ≥ 25 dB HL) vs. consideration of those with *a moderate or greater* hearing loss (PTA > 40 dB HL) as the hearing-impaired group likely constitutes different comparison groups and underlying idea of what is a hearing “loss” across different studies.

Varied measures of cognition used have also placed challenging demands on synthesis of prior results. A plethora of validated neurocognitive assessments exist both across cognitive domains and as global screeners. Variation in domain, mode of administration, if accommodations for hearing loss were considered, whether cognitive status was determined by provider or proxy report exist across studies, among others. While the importance of evaluating cognitive ability in multiple ways should not be ignored due to the complexity of cognitive processing and complex diagnostic presentation, synthesis of results across studies remains aloof.

### Central Auditory Function

While an association between cognitive decline/dementia and peripheral auditory function is well established (Livingston et al., 2020), the relationship between cognitive decline/dementia with central auditory function has been less studied in the epidemiologic literature. Given the interdependence between central auditory function and cognitive processing, it becomes difficult to distinguish between the two processing abilities. For speech to be properly understood, the auditory stimuli must first maintain sufficient integrity to be accurately encoded by the peripheral auditory system and must then be decoded by the central auditory system (Humes, 2021). For the auditory signal to be decoded by the central auditory system, higher level cognitive processing is required. Research over the last few decades has attempted to distinguish the boundaries between central auditory dysfunction (CAD) and cognitive impairment (CHABA, 1988; Humes et al., 2013a). Heterogeneity in defining central auditory function and cognitive processing has led to significant barriers to evidence synthesis and causal inference. As with peripheral hearing loss, a diverse array of definitions, tests, cognitive domains, and sample populations has been utilized for studies across disciplines.

Prior systematic reviews have revealed mixed quality and potential bias in previous work on central auditory function and cognition (Dryden et al., 2017). Dryden et al. (2017) report an overall weak correlation between cognitive performance and speech perception/central auditory function depending on the cognitive domain and speech-in-noise assessment used. The strongest correlation was observed between central auditory function and processing speed. It has yet to be determined what degree un-aided peripheral hearing ability has in the moderating or modifying of the association between central hearing (e.g., decoding of speech stimuli or speech perception ability in noise) and cognitive performance. Overall, results demonstrate an interconnection in cortical resources utilized for both processes (Humes, 2013; Humes et al., 2013b; Humes and Young, 2016; Sardone et al., 2019), presenting challenges for inference but also noteworthy opportunity to identify novel targets for intervention or prevention.

It has been hypothesized CAD may serve as an early marker and therefore a possible prodromal symptom of cognitive decline (Gates et al., 1996). We know AD pathology reaches the auditory cortex. Nearly 30 years ago, Sinha et al. (1993) demonstrated the presence of plaques and neurofibrillary tangles within the medial geniculate body and central nucleus of the inferior colliculus in patients diagnosed with AD compared to those without a diagnosis. Additionally, neurofibrillary plaques and tangles were found in the primary auditory and auditory association cortices of patients diagnosed with AD. While, Braak staging (Braak and Braak, 1991, 1997) suggests these auditory cortices are some of the final brain regions affected by AD pathology. However, evidence indicates functional disconnection effects from the involvement in higher order cortical areas may affect the primary auditory cortex in early Alzheimer’s disease (Sardone et al., 2019). Importantly, the cortical areas associated with age related hearing loss and compensatory cortical activation to support language processing like the cingulate cortex are also involved in cognitive function like episodic memory and are affected early in the AD disease process, having specific implications for complex auditory processing (Goll et al., 2010, 2011, 2012; Sardone et al., 2019). These ideas support CAD as a marker of AD, but with unclear indication of CAD as an *early* marker (Sardone et al., 2020). How this presentation of pathology at later stages of progression translates to earlier identification of AD, beyond abilities from currently identified neurological and biological biomarkers is unclear. Yet behavioral testing of central auditory function suggests utility. Gates et al. (2011) demonstrated in a sample of volunteers from a dementia surveillance cohort over nine times greater hazard for incident dementia (95% CI: 3.6, 26.7) among those with severe CAD compared to those with proposed normal function. A pilot study in a well-characterized aging cohort found poor cognitive performance on memory, language, executive function, and global function for those with poor speech-in-noise performance regardless of peripheral hearing status (Mamo et al., 2019). The associations for some domains were attenuated after excluding those with a moderate or greater hearing loss, suggesting peripheral hearing may play a role in performance within certain cognitive domains that are dependent on speech tasks.

Many prior studies have employed the Dichotic Sentence Identification test (DSI) as a measure of central auditory function. The DSI presents a meaningless but grammatically correct sentence to each ear simultaneously and the listener is to then identify the sentences, with designated systematically differing modes of reporting the sentence items (Fifer et al., 1983). This diagnostic tool, primarily used currently in research settings, was designed to be less susceptible to peripheral hearing ability when presented at a conversational level of 50 dB HL. Therefore, some propose poor performance on the DSI test may be more reflective of cognitive ability rather than incorporating influence from peripheral hearing and the simple inability to hear the test (Jerger, 2019). DSI has been used to differentiate memory impairment among those demonstrating mild memory impairment without dementia (Gates et al., 2008). However, the DSI test and many other central auditory function assessments have minimal clinical utility due to complex presentation requirements and time-intensiveness.

Investigation of various means to define and measure central auditory function suggest an association with subjective memory complaints/impairment (Idrizbegovic et al., 2011; Fausto et al., 2018). In a small sample, subjective memory complaints were associated with poor performance on certain central auditory processing evaluations but not with peripheral hearing ability (Jayakody et al., 2020), potentially further supporting speech-in-noise difficulty as a marker of cognitive function. Yet proper implementation of this information for dementia prevention and intervention remains unclarified.

Across measures of central auditory processing, an association has been observed between biomarkers such as CSF tau, cortical thickness and volumetric measures of AD-related brain regions (Tuwaig et al., 2017), although existing supporting evidence remains limited. Given the rapidly expanding identification of dementia related biomarkers, what central auditory processing measures may add to our existing repertoire is an important consideration for future research and identification of pre-clinical dementia, whether considering generic ‘dementia’ and all subtypes, or considering more targeted research on AD specifically given its epidemiologic importance. Consideration of sensory loss more broadly (i.e., hearing, vision, and olfactory ability) has received more recent attention as indicators of pre-clinical dementia. For example, advances in retinal imaging may shed light on early brain changes associated with Alzheimer’s disease (Snyder et al., 2016; Zhang et al., 2021). Yet, like central auditory processing, there is much we still must determine regarding these measures and neurological pathways to understand any potential role of sensory biomarkers in identification of preclinical dementia.

### Dementia Prevention Through Management of Hearing Loss

Due to low-risk auditory rehabilitation tools such as hearing aids, hearing loss in older adults is readily considered modifiable and therefore makes it a worthy target of interest among dementia risk factors. The current limited use of hearing aids and challenges with accessibility of hearing devices and services have been a focus of both health research and public policy in recent years (National Academies of Sciences, Engineering, and Medicine, 2016), bringing new force behind the idea of hearing loss management as a means for dementia intervention. Existing data and reports of the use of hearing aids for cognitive decline stem largely from observational and cross-sectional study and present mixed findings (Amieva et al., 2015; Maharani et al., 2018; Ray et al., 2018; Dawes et al., 2019; Amieva and Ouvrard, 2020). In observational studies, we are often not able to completely control for factors which determine or influence if an individual pursues or uses hearing aids or other forms of hearing treatment (e.g., socioeconomic status, education background, social support and engagement, access to healthcare) — many of these factors are also protective against cognitive decline. These challenges present difficulty when trying to isolate the potential protective effect from hearing aids from that of these external factors, potentially influencing estimated effects due to aspects of selection into study of hearing aid use. Well-designed pilot interventions and clinical trials have taken up this challenge in recent years to determine if the use of hearing aids may alter subsequent risk of dementia (Brewster et al., 2020). These clinical trials present unique opportunity inherent within study design by minimizing this potential bias from confounding factors through design tools like randomization and masking (Celentano and Szklo, 2020). One of the largest and longest clinical trials to date on the use of hearing aids for dementia prevention is currently underway in The Aging and Cognitive Health Evaluation in Elders (ACHIEVE) clinical trial (Deal et al., 2018). While not powered to detect a difference for memory scores, the ACHIEVE pilot study demonstrated suggestion of an efficacy signal in memory test performance for participants randomized to hearing treatment, including hearing aid use, compared to a health education control (Deal et al., 2017). The pilot study also indicated positive proximal outcomes of improved participant perception of hearing handicap and improved social network. We have yet to determine how age and rapidity of hearing loss onset and its proximity to hearing aid uptake may modify dementia risk. The influence of other rehabilitation strategies on dementia risk, such as basic communication strategies, cochlear implants, or auditory training, is vastly understudied.

## Potential Pathways Linking Hearing to Cognition

**FIGURE 4:**
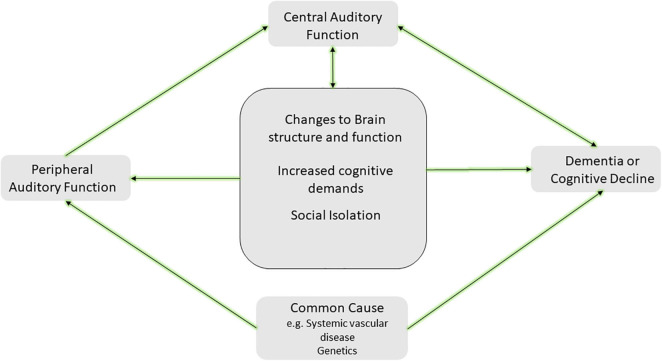
Hypothesized framework for the mechanism of the hearing and dementia association. The center square includes potential causal paths between peripheral hearing loss and cognitive decline or dementia, including changes to brain structure and function (i.e., sensory deprivation hypothesis), increased cognitive demands (i.e., information degradation hypothesis), and other effects such as social isolation. Additionally, a common cause such as systemic vascular disease or genetic factors may lead to both peripheral hearing loss and cognitive decline and dementia. Further inclusion of central auditory function resulting from direct and indirect effects of this causal pathway is depicted and may serve as a marker of cognitive decline or dementia. It is likely more than one of the pathways depicted may explain the link between hearing and dementia. Adapted from Powell et al. (2021).

The complexities and interdependence between central auditory function and cognitive processing present challenges for causal inference. Therefore, current hypotheses of mechanistic pathways predominantly view peripheral hearing loss as a potential cause of dementia and consider central auditory function as a marker of cognitive decline. Current hypotheses of the causal mechanistic pathways have been well articulated in other works (Lin and Albert, 2014; Wayne and Johnsrude, 2015; Griffiths et al., 2020; Johnson et al., 2021; Powell et al., 2021). Clarifying this mechanistic pathway has far-reaching implications for research, the design, implementation, and evaluation of dementia prevention strategies, and the translation of the evidence to recommendations for patient care or health policy. A smaller body of evidence currently supports the notion that dementia leads to hearing loss (CHABA, 1988; Baltes and Lindenberger, 1997). A larger collection of evidence supports the notion hearing loss may contribute toward cognitive decline— directly through what is commonly known as the sensory deprivation hypothesis, or indirectly through the information-degradation hypothesis (Wayne and Johnsrude, 2015). Alternatively, hearing loss and dementia may result from an additional external factor through what is considered the common-cause hypothesis (Wayne and Johnsrude, 2015; Powell et al., 2021). In this article, we briefly review evidence to support each hypothesized pathway as depicted in our modified mechanistic framework (Figure 4).

### Sensory Deprivation

A growing body of evidence indicates prolonged sensory deprivation has an adverse effect on the brain, with enduring changes to brain structure and function. Due to auditory deprivation from ARHL, neural deafferentation, cortical re-allocation for support of other processes, and atrophy in brain regions important for speech perception processing may occur. The resultant reorganization may add to existing brain pathology (e.g., amyloid burden, neuronal loss) at the detriment of general cognitive performance by altering critical brain regions which could otherwise be utilized for higher-level cognitive processing. Reduced gray matter density and reductions in temporal lobe volume are observed in those with peripheral hearing loss (Lin et al., 2014; Wingfield and Peelle, 2015; Armstrong et al., 2019), as prolonged sensory deprivation may lead to changes and decreases in cortical brain volume in conjunction with that seen with expected cognitive aging or neurodegenerative disease (Lin et al., 2014; Eckert et al., 2019). The brain regions most notably affected are pertinent for semantic memory and preclude advancement along the continuum of cognitive decline, including frontal and pre-frontal regions, the superior temporal gyrus and Heschel’s gyrus (Husain et al., 2011). Cortical regions enlisted for speech understanding like the frontal cortex and hippocampus are also associated with reductions in whole-brain gray matter volume (Rudner et al., 2019). Even among those older adults considered free of dementia, hearing loss appears to be associated with reduced white matter microstructural integrity in cortical regions crucial for cognitive processing (Rigters et al., 2017; Alfandari et al., 2018; Armstrong et al., 2019; Croll et al., 2020). Research has yet to determine importance of the duration of sensory deprivation or evidence for a sensitive period in age of onset of hearing loss and how this relates to the structural changes observed. While cortical changes and reorganization from hearing loss is evident even among those in the earliest stages of cognitive impairment (Campbell and Sharma, 2014; Sharma and Glick, 2017), the extent of cortical reorganization necessary to evoke brain atrophy and changes to cognitive performance is unclear. Prolonged sensory deprivation, therefore, may result in physical reductions in cortical volume in addition to that experienced from dementia pathology alone. This sensory deprivation may necessitate cortical reorganization resulting in further restriction of cortical capacity available for cognitive processing. How and if these brain volume changes represent specific pathologic changes for types of dementia remains to be clarified. Longitudinal study of the role of peripheral hearing loss and association with AD amyloid pathology on PET imaging did not suggest an association (Parker et al., 2020) but work in this space using specified biomarkers of dementia has been understudied.

### Information Degradation

While the sensory deprivation hypothesis suggests physical changes to the brain due to prolonged hearing loss which subsequently impacts cognitive processing, the information degradation hypothesis proposes the association stems from increased demands on cognitive processing in order to compensate for impoverished sensory input. The additional processing required to compensate for degraded auditory perception draws on the same resources needed for other higher-level cognitive processing and semantic encoding (Peelle, 2018). These increased cognitive demands required to appropriately encode auditory stimuli requires extensive listening effort particularly with memory, attention, and executive function (Wingfield et al., 2005; Tun et al., 2009, 2012; Pichora-Fuller et al., 2016; Peelle, 2018). An individual’s ability to compensate for subsequent diminished cognitive performance is heterogeneous and may contribute toward the known variability in cognitive performance, presentation, and clinical symptoms among similar existing brain pathology. With hearing loss, this increased cognitive and listening effort may increase cognitive load at any time of day as the auditory system never “turns off,” thereby drawing on the cognitive buffer of the individual and their ability to compensate for cognitive changes and resulting in earlier presentation of clinical symptoms and dementia (Whitson et al., 2018). The compensatory mechanisms activated on cognitive tasks between older and younger adults with sensory deficits differ, with older adults demonstrating increased fatigue on dual tasks that rely on both listening and understanding (Schneider et al., 2002). Essentially, the information degradation hypothesis implies acute cognitive impairment. If we are able to restore auditory input, we may therefore see at least some restoration of cognitive performance on higher-level tasks.

### A Common Cause of Both Conditions

Alternatively, both hearing loss and cognitive impairment may merely stem from the same underlying mechanism (Baltes and Lindenberger, 1997; Wayne and Johnsrude, 2015). Given the presumed independent effect of peripheral hearing on cognitive processing when measured through audiometry, investigation of a common cause which may be associated with hearing and cognition has largely been evaluated via pure tone audiometry. A potential common mechanism might include the overall neural degeneration common with aging, resulting in both decreased auditory ability and decreased cognitive performance (Surprenant and Neath, 2006; Wayne and Johnsrude, 2015). Additionally, a given dementia pathology (most commonly discussed as AD pathology) may lead to early CAD and impaired hearing prior to more general cognitive decline (Johnson et al., 2021). Decreased processing speed with age may lead to slower cognitive functioning overall as well as slower processing speed for sensory integration and perception (CHABA, 1988; Salthouse, 1996). Dementia pathology from a central cause may therefore produce early dysfunction within the central auditory system resulting in impaired hearing ability (i.e., auditory scene analysis or ability in complex listening environments) prior to the presentation of more general cognitive decline or impairments. As described in the review by Griffiths et al. (2020), it is also possible degraded peripheral signals lead to dysfunctional pattern processing within the central auditory system (particularly the medial temporal lobe) and interacts with existing neurodegenerative or AD pathology, resulting in the observed association. With this hypothesis, whether the increased neuronal activity from degraded auditory input causing or increasing AD pathology, or the degraded auditory input from hearing loss may reduced activity for auditory cognitive processes within the medial temporal lobe remains to be clarified, however, either speculation supports early identification of hearing loss prior to prolonged duration of decreased auditory input and broad hearing-dementia intervention strategies.

It is possible both auditory and neural functioning are altered by systemic vascular pathology impacting the spiral ganglion or stria vascularis of the auditory system and bran vasculature. Therefore, vascular disease in older adults may also subsequently lead to concurrent cognitive and sensory changes (Eckert et al., 2013; Laughlin et al., 2013; Livingston et al., 2017), although this hypothesis is challenging to confirm due to the long prodromal stage of some dementias like AD. Exploration of a common genetic risk between AD and hearing loss using a weighted sum of AD polymorphisms and AD inflammatory pathway suggests a possible genetic link with poorer speech-in-noise performance and self-reported difficulty hearing in background noise (Brenowitz et al., 2020). However, study of Apolipoprotein ε4 (*APOE* ε4) has shown no association with hearing loss (Morita et al., 2019), nor does it modify the association between hearing loss and cognitive decline (Alattar et al., 2020). Other work suggests the presence of one or more *APOE*ε4 alleles may actually be marginally associated with better hearing in older adults as measured via pure tone audiometry, perhaps because *APOE* ε4 is more common in Black adults, but hearing loss is more prevalent in White adults (Mener et al., 2016). While *APOE* ε4 serves as a strong risk factor for dementia, based on current evidence, it does not appear to be a driving factor in the hearing-dementia association. Although the genetic determinants of ARHL are largely unknown, identifying what (if any) genetic determinants exist may provide further insight toward additional avenues of a common cause between hearing loss and dementia (Wells et al., 2019). Overall, few studies have investigated the role of genetic risk on causality in the hearing-dementia link, therefore some question of association with shared genes remains (Mitchell et al., 2020).

### Potential Mediators of the Hearing Loss-Dementia Association

Other risk factors for dementia may also mediate the association of hearing and cognition including: loneliness, social isolation, depression, decreased physical activity, or frailty. Studies have demonstrated the presence of hearing loss leads to increased social isolation and/or loneliness for some older adults as individuals with hearing loss may withdraw from previously enjoyed activities or social interactions due to the inability to engage and communicate with others. Links between social isolation or loneliness and dementia are well established (Rutherford et al., 2018; Maharani et al., 2019; Rafnsson et al., 2020; Shukla et al., 2020). The evidence for link between hearing loss and depression, which may serve as a risk factor and/or prodromal sign of dementia, remains mixed (Rutherford et al., 2018). While an increasing body of evidence suggests increased risk of depression with hearing loss, the direction of association remains to be clarified. The heterogeneous course of depression in older adults as it relates to hearing loss has received minimal investigation (Brewster et al., 2018). It is possible hearing loss leads to reduced physical activity and physical functioning, each a known risk factor for dementia, due to decreased auditory awareness with surroundings and anxiety (Chen et al., 2015; Choi et al., 2016; Martinez-Amezcua et al., 2021). Some evidence suggests a greater prevalence of frailty among those with hearing loss, another condition which has been suggested as an independent risk factor or early marker of dementia (Panza et al., 2015; Liljas et al., 2017). Each proposed potential mediator and pathway presents an identified opportunity for targeted intervention, yet how each may fit within a larger dementia framework remains to be clarified.

### The Challenges of Multiple Potential Pathways

Multiple potential pathways area likely involved in the association between hearing and dementia, but with unknown understanding of the specific contribution of each pathway and if this contribution varies by the individual. While common risk factors (i.e., age, education, vascular disease) are thought to contribute toward the association (Whalley et al., 2004; Humes, 2013; Wayne and Johnsrude, 2015), these factors likely do not explain the full story. Even in large epidemiologic studies which have attempted to control for these additional factors, the association persists, suggesting additional pathways are likely involved but have yet to be differentiated.

## Targeted Future Research

**FIGURE 5:**
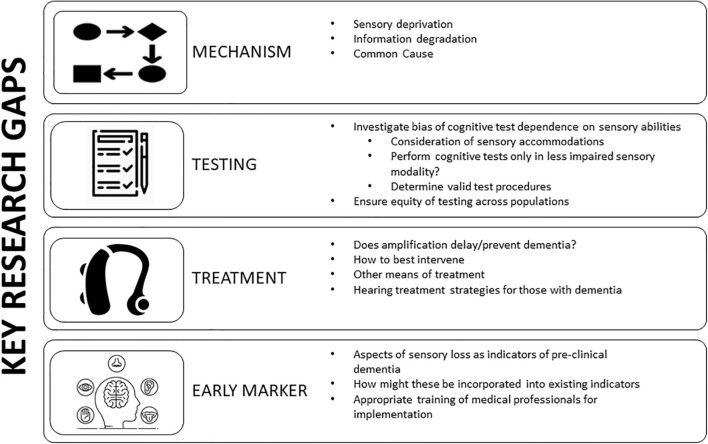
Key research gaps in hearing impairment and cognition. Targeted research on the mechanism behind the association between hearing and cognition will guide intervention and prevention strategies. Additional research gaps include an understanding of potential bias in cognitive testing due to dependence on sensory abilities and how best to study and minimize this bias. Further understanding of if treating hearing loss via amplification or another means influences dementia risk is vital for public health. Determination if markers of sensory impairment may serve as indicators of pre-clinical dementia and how this may be incorporated with existing biomarkers. Adapted from Powell et al. (2021).

Identification of research priorities given the transdisciplinary scope of study on hearing and dementia will aid in achieving foundational dementia research goals: to advance our identification of subgroups of adults who may present greater risk for cognitive decline/dementia or suggest novel options for intervention or prevention. Numerous pressing research questions and additional perspectives on the importance of these questions prevail (Wayne and Johnsrude, 2015; Whitson et al., 2018; Powell et al., 2021). Here we feature four research priorities which, in our opinion, will provide leading advancements in the possible causal role of hearing loss along the dementia continuum: (1) determination of the pathway of association, (2) management of potential bias due to sensory loss in cognitive testing, (3) determining if we can modify dementia risk by treating hearing loss, (4) understanding if biomarkers or indicators of sensory loss are useful for identification of pre-clinical dementia (Figure 5).

### By What Pathway Does the Hearing-Cognition Association Act?

Given the varied temporal contributions of each proposed mechanistic pathway, determination of which pathway(s) serve as the primary driver behind the association is vital. This determination may allow for improved study design and planning and well as more informed clinical intervention to delay or prevent the onset of cognitive symptoms and delay the progression along or alter the trajectory of the clinical course of dementia. With the current insufficient evidence to declare hearing loss has an independent effect on cognitive impairment for older adults, how we should intervene on hearing loss, when to do so, and if such interventions are effective in decreasing or delaying dementia risk will all largely depend on the underlying mechanism. We know the presentation of dementia symptomatology is heterogeneous across individuals even with the same underlying pathology. We have yet to determine by what manner hearing loss may influence a potential buffer which delays/prevents the presentation of symptoms in some individuals yet fails do so in others, leading to earlier symptomatology.

If the association between hearing loss and dementia is only due to a common cause, the potential for directly delaying or preventing changes in the presentation of dementia symptoms or pathology through hearing would be extremely limited as the underlying cause would remain untreated. The research intervention focus would ultimately turn toward the underlying common confounder, which could then have downstream effects for hearing and cognition. However, the use of hearing management such as hearing aids may support cognition through indirect pathways like improving social engagement and communication yet decreasing cognitive load and strain on central auditory function through improvement of peripheral auditory input, directly preventing dementia would remain elusive.

A direct effect of hearing loss on cognitive function is inferred with the sensory-deprivation and information degradation hypotheses. A long-term versus short term-effect is implied depending on the proposed pathway, yet both advocate that hearing loss, and notably untreated hearing loss, leads to downstream consequences for cognitive resources with important implications for approaches to intervention. The sensory deprivation hypothesis posits structural and functional changes in the brain due to lack of auditory stimulation. Therefore, preemptive identification of hearing loss and early adoption of treatment strategies which delay or prevent deafferentation or atrophy within the auditory or sensory regions of the brain are essential to prevent lasting structural damage. Importantly, auditory rehabilitation to prevent structural and functional changes in the brain through sensory deprivation may even have benefit at later stages of hearing loss by delaying additional neural reorganization or atrophy. For comparison, the information degradation hypothesis strongly supports hearing intervention at any time in the disease process. With restoration of the integrity of auditory stimuli via rehabilitation or hearing management, the cognitive demands and utilized cognitive resources for speech and environmental awareness would in theory be reduced, allowing for cognitive resources to again be allocated to overall cognitive processing and compensation.

It is imperative we determine how to disentangle CAD and cognitive decline if we are to consider its use in targeting prevention efforts or early identification of subgroups at greater risk for cognitive decline. How to differentiate between changes in cognitive processing and CAD, the temporality between each processing condition, and whether CAD may serve as an early marker for specific subtypes of dementia remains a challenging barrier for characterizing the link between hearing loss and dementia. While the majority of research to date has focused on the association between hearing loss and Alzheimer’s disease since the most common form of dementia and pathology observed, distinctions in association with other dementia pathologies is paramount. The dependence and entanglement of both forms of hearing on higher-level cognitive functioning stirs question of how to interpret the role of each process along the auditory pathway. If CAD may serve as an early marker of dementia remains to be determined.

As stated, it is likely multiple mechanistic pathways are involved in the hearing-dementia association. Determining if one pathway serves as the primary driver, or if the pathway is unique to the individual may provide an opportunity for a person-centered approach to dementia prevention and intervention. Furthermore, while not mutually exclusive, determining the role of central vs. peripheral hearing in the hearing-dementia association will further inform research and clinical use of hearing and prevention or as an early marker of dementia.

### How Do We Manage Potential Bias Due to Sensory Loss in Cognitive Testing?

A goal of any neurocognitive test battery is to accurately measure cognitive function for all individuals. Yet the validity of cognitive assessments inherently depends on an individual’s auditory or visual ability to access test materials. This interdependence has led some to question whether there is a bias or confounding introduced by a sensory impairment during cognitive testing for older adults (Guerreiro and Van Gerven, 2017; Füllgrabe, 2020). An investigation testing this hypothesis used item response theory methods to disentangle the true effect of sensory impairment on test performance (Nichols et al., 2021) in two longitudinal aging cohorts. Results suggest some differences in performance related to sensory loss, particularly among those with the poorest health or greatest cognitive impairment. However, for the vast majority of participants, cognitive performance on testing was not found to be biased due to sensory impairment (Villeneuve et al., 2017; Bott et al., 2019, 2020). Further determination of how best to conduct cognitive testing among those with sensory loss is vital for clinical and research settings.

While exclusion of those with severe sensory loss from testing might ensure completion of evaluations, this exclusion limits our understanding of the role of sensory loss in cognition. One way to assess cognitive function among those with hearing loss may be to utilize cognitive test batteries that are administered via visual modality rather than auditory. However, this strategy does not fully address issues that arise among those with dual sensory impairment (i.e., those with both vision and hearing loss).

An alternative approach to address sensory loss when administering cognitive assessments is to consider accommodations for the sensory loss (Littlejohn et al., 2021). If an individual usually wears a hearing aid or glasses, it would be important to remind the individual to bring these sensory aids for optimal completion of evaluations. Accommodations for sensory loss might include offering test instructions in written (i.e., large print) as well as oral format, ensuring the testing is completed in a quiet and well-lit environment, or provision of a personal amplifier such as a pocket-talker or a visual aid for test takers. Accounting for hearing and vision needs during study conception, design and analysis, as well as during test administration will be important for confidence in study results. Regardless of the different types of cognitive test used, it is imperative that we include individuals with different types and degrees of sensory loss in studies, in order to advance our understanding of the role of sensory loss in cognition (Pye et al., 2017; Littlejohn et al., 2021).

### Can We Modify Dementia Risk and Its Associated Behaviors by Treating Hearing Loss?

Recent decades have seen an accumulation of evidence for the impact of treating hearing loss on cognitive decline or dementia. Our observational studies have provided foundational and pivotal insights into the hearing-dementia association, however, evidence of causality for decreased or delayed cognitive decline due to hearing aid use is more challenging. From a neurocognitive perspective on this investigation, challenges include selection effects of older adults into studies. Individuals who pursue and obtain a hearing aid are generally a select group of adults— often with higher education, higher income, and greater health seeking behaviors— all of which are considered protective factors for cognitive decline (National Academies of Sciences, Engineering, and Medicine, 2016; Livingston et al., 2020; Reed et al., 2021).

Many barriers to obtaining hearing aids still exist within the United States and must be considered within the context of existing research on the use of hearing aids as dementia prevention or intervention. Hearing aid and aural rehabilitation services are not covered under Medicare or many private insurances, placing the cost of the device and services (often $1000 or more) on the individual and family. Further, an audiologic evaluation and aural rehabilitation appointments are not currently available through direct-access to patients under some American insurances, but require referral from a primary care physician or specialist (National Academies of Sciences, Engineering, and Medicine, 2016). This added barrier with a need for both patients and providers to understand how to navigate the healthcare system and for patients to attend additional medical appointments currently creates added barriers to hearing treatment which may prevent or deter some individuals from seeking care. While access to hearing devices and services is improved in some countries, global barriers remain as far as availability of trained professionals or cost of devices (World Report on Hearing, 2021). Additional challenges include collection of hearing data and data on hearing aid use, and unmeasured confounding. Data on hearing ability is often not collected or collected only in very limited form in many epidemiologic or non-auditory based studies. Additionally, data on hearing aid use and amount of use is virtually non-existent. Owning a hearing aid is not synonymous with using a hearing aid. Further, individuals vastly over-report their use of the hearing aid (Taubman et al., 1999), yet wearing a device regularly and often is the only means to ensure the brain has had the ability to adapt to increased auditory stimuli. What constitutes “regularly and often” remains to be clarified and may be individualized (Hickson et al., 2014). Without this information, it remains challenging for us to determine how hearing aid use may modify the hearing-dementia association, or any other health outcome for older adults.

Without understanding the mechanism(s) behind the hearing-dementia association, it is challenging to determine how and when is best to intervene on cognitive decline and dementia. Evidence for treatment of hearing loss throughout the dementia continuum is growing, yet many questions remain. Evidence of efficacy, effectiveness and cost efficiency will aid in determining the best aural rehabilitation choices for prevention — all areas with extensive gaps in evidence for hearing loss and dementia.

In addition to potentially serving as a dementia prevention strategy, hearing aids may present opportunity for intervention for neuropsychiatric symptoms (NPS) at all stages of dementia and negative health outcomes associated with NPS (Kales et al., 2014). Breakdowns in communication between those individuals with dementia/cognitive impairment and their caregivers along with decreased awareness of surroundings may contribute to behavioral changes commonly noted with more progressed disease (Littlejohn et al., 2021). The use of a hearing assistive device in older adults with cognitive impairment has been found to result in fewer and less severe NPSs and less severe depressive symptoms (Kim et al., 2020). Therefore, hearing aid use among dementia patients demonstrate improved communication, reduced NPS, decreased perception of hearing handicap, and improved quality of life for both individuals and their caregivers (Dawes et al., 2015, 2019; Mamo et al., 2018; Littlejohn et al., 2021).

Additional work is needed to determine how best to provide auditory rehabilitation for adults with cognitive impairment. A recent study (Hubbard et al., 2018) suggests treating hearing loss in cognitively impaired older adults yields benefits for both the older adult as well as caregiver dyads. A recent internationally gathered task force on identification and management of hearing and vision loss among those living with dementia (Littlejohn et al., 2021) highlighted six key areas of practice recommendations and identified targeted areas for improvement, including: awareness and knowledge, recognition and detection, evaluation, management, support, services and policies. However, the group acknowledged that a dearth of evidence across many of these areas left the basis of broad recommendations to be placed on expert and patient input at times, vowing to revisit recommendations in a few years in the hopes of improved scientific evidence. Even with expert recommendations for management, hearing is not automatically “restored” with the use of a hearing aid. Instead, hearing aids should be considered a tool to improve audibility and access to environmental and speech sounds. For a new hearing aid user, most individuals require an adjustment period as the brain re-habituates to increased access to the auditory environment and stimulation after a potentially prolonged period of sensory deprivation. This aspect of hearing loss management may be unclear or unknown to patients and non-auditory science researchers without the appropriate training and counseling. How to provide this counseling for persons with dementia and their families has, to our knowledge, not been studied.

While hearing aids serve as the most prominent means of hearing intervention, other means of aural rehabilitation are available such as personal amplifiers, general assistive listening devices, or auditory training strategies. Research is limited on the use of these alternative strategies for treatment of hearing loss along the dementia continuum, yet determination of a variety of accessible alternative strategies is fundamental for providing communication ability and social engagement for older adults. Hearing aids may not be feasible for some older adults (e.g., those with severe cognitive impairment) and likely do not meet the listening needs of many adults when used as the only form of rehabilitation. As mentioned, adaptation to increased auditory stimuli requires extensive cortical adjustment which may overwhelm some individuals without proper counseling and consideration. Non-technology driven alternative strategies (e.g., training in communication strategies such as speaking face-to-face, turning off or reducing the presence of competing background noise, talking from the same room, use of additional visual cues/aids, improving self-efficacy advocacy) (Nieman et al., 2017) require further evaluation to determine how these proven strategies might be utilized for older adults with cognitive impairment.

Importantly, the use of hearing aids has a primary role in management of peripheral hearing loss, contributing to the focused effort of public health research on peripheral rather than central hearing ability and the association with cognitive decline/dementia. If the pathway linking hearing to cognition is related (directly or in part) due to central auditory processing and ability, the use of hearing aids as a prevention and intervention option for dementia will have minimal effect given the state of current technology and auditory processing treatments. While hearing aid use may not delay the progression of dementia pathology if the association is due to a common cause, as stated above the use of hearing aids may still indirectly support intervention of progression of the clinical presentation of dementia symptoms through indirect pathways such as decreased cognitive load and improved social stimulation. Future advances in hearing aid technology which may better address cognitive and central auditory demands may provide even greater benefit. Currently, evidence for the role of central auditory processing and central hearing disorders for early identification of dementia will have heightened importance and emphasize the need for consideration of greater use in neuropsychological and medical evaluations.

### Can Sensory Loss Be an Additional Tool for Identification of Pre-clinical Dementia?

The high prevalence of not only hearing loss, but also vision loss, among older adults presents opportunity for additional access points for identification of pre-clinical dementia *if* hearing or vision may serve as an early marker of dementia- a point which has yet to be determined and has received minimal study (Brenowitz et al., 2019; Johnson et al., 2021). We do not yet know if sensory loss indicators (i.e., optical coherence tomography, fundus photography, speech-in-noise testing, auditory scene analysis or auditory cognitive testing, electrophysiology, etc.) aid in earlier identification of cognitive change. If sensory loss *does* prove to be a valuable tool for identification of pre-clinical dementia, then we must determine how to use this tool in patient-provider planning and diagnosis as well as within our research models. Additionally, training on discussion with health care professionals for the appropriate referrals and how to convey results within each represented scope of practice is essential for appropriate dementia planning (Littlejohn et al., 2021).

## Conclusion

Although research on the role of hearing loss on dementia risk initiated over 40 years ago, recent surge in the evidence supporting adverse outcomes across health and care among older adults with hearing loss has come to the attention of patients and their families, clinicians, and policy makers. The human and economic expense of caring for the expected growing number of adults reaching or past retirement age demands interdisciplinary collaboration. While the fields of cognitive science, neuroscience, and gerontology have been at the core of dementia research, allied-health disciplines such as auditory science present untapped opportunity to contribute toward efforts to reduce the burden of dementia across societies. A thorough understanding of the current evidence, identification of leading hypothesis and elucidation of the mechanism(s) driving the association between hearing and dementia, consideration of sensory loss during cognitive testing or in care strategies for those with dementia or dementia prevention, and targeted future directions of research can have a significant impact on prevention and intervention strategies for older adults. The interdependent and synergistic processes of hearing and cognition require careful approach for research and clinical care. The current limitations in dementia treatment necessitate innovative approaches for dementia intervention. Identification of additional early markers of dementia, a potential role of central auditory function with continued research may aid in this process, as well as inform optimal strategies for hearing management itself. Considering the presence of other conditions, like hearing loss, and optimizing our strategies to evaluate and treat hearing loss could diminish the risk of adverse outcomes and enhance health and quality of life for older adults.

## Author Contributions

DP was responsible for the initial drafting, editing, and formatting. EO, NR, FL, and JD contributed to content and editing. All authors contributed to the article and approved the submitted version.

## Conflict of Interest

NR reports being a scientific advisory board member of Shoebox Inc and Neosensory. FL reports being a consultant to Frequency Therapeutics, speaker honoraria from Caption Call, and being the director of a public health research center funded in part by a philanthropic gift from Cochlear Ltd to the Johns Hopkins Bloomberg School of Public Health. The remaining authors declare that the research was conducted in the absence of any commercial or financial relationships that could be construed as a potential conflict of interest.

## Publisher’s Note

All claims expressed in this article are solely those of the authors and do not necessarily represent those of their affiliated organizations, or those of the publisher, the editors and the reviewers. Any product that may be evaluated in this article, or claim that may be made by its manufacturer, is not guaranteed or endorsed by the publisher.
